# Hominy

**Published:** 1854-03

**Authors:** 


### HOMINY.

It is surprising how little is known of this nutritious healthy food, and what an excellent substitute it is for potatoes during the continuation of the disease among them, which renders some that are fair to the eye, unfit for food, and all exceedingly dear. As we write, our hostess informs us that potatoes, hominy, and white beans are all of the same price—$2.50 a bushel, and rice but a little dearer. If a man can afford to eat fried gold for breakfast, boiled bank notes for dinner, and roasted dollars for supper, he can afford to eat potatoes cooked in the same way, and not otherwise, at present prices. In point of economy as human food, one bushel of beans or hominy is equal to ten of potatoes. Hominy, too, is a dish almost as universally liked as potatoes, and at the South, about as freely eaten, while at the North it is seldom seen. In fact, it is an unknown food, except to a few persons in cities. By hominy we do not mean a sort of coarse meal, but grains of white corn, from which the hull and chit, or eye has been removed, by moistening and pounding in a wooden mortar, leaving the grains almost whole, and composed of little else but starch. It has often been said, not one cook in ten knows how to boil a potato. We may add another cypher when speaking of the very simple process of cooking hominy. We give the formula from our own experience, and instructions received in a land where "hog and hominy" are well understood.—Wash slightly in cold water, and soak twelve hours in tepid, soft water, then boil slowly from three to six hours in the same water, with plenty more added from time to time, with great care to prevent burning. *Do not salt while cooking*, as that or *hard water* will harden the corn. So it will peas or beans, green or dry, and rice also. When done, add butter and salt; or a better way is to let each season to suit the taste. It may be eaten with meat in lieu of vegetables, or with sugar or syrup. It is good hot or cold, and the more frequently it is warmed over, like the old-fashioned pot of

> "Bean-porridge hot, or bean-porridge cold,
> Bean porridge best at nine days old."

So is hominy—it is good always, and very wholesome, and like tomatoes, only requires to be eaten once or twice to fix the taste in its favor.

Hominy Breakfast Cakes.—Mash the cold hominy with a rolling-pin, and add a little flour and milk batter, so as to make the whole into little cakes in the hand, or it may be put upon the griddle with a spoon. Bake brown, eat hot, and declare you never ate anything better of the batter cake kind.

Hominy and Milk, hot or cold, is as much better than mush and milk, as that is better than oat-meal porridge.

Hominy Pudding.—Prepare as for batter cakes. Add one egg to each pint, some whole cinnamon, sugar to suit the taste, and a few raisins, and bake like rice pudding. A little butter or chopped suet may be added. Serve hot or cold, with or without sauce.

Hominy and Beans.—Mix equal parts of cold baked beans and hominy together, and heat up, and you will have an excellent dish.—*The Plow.*

